# Cluster classification of a Brazilian gastric cancer cohort reveals remarkable populational differences in normal p53 rate

**DOI:** 10.31744/einstein_journal/2024AO0508

**Published:** 2024-09-20

**Authors:** Fábio Ribeiro Queiroz, Letícia da Conceição Braga, Carolina Pereira de Souza Melo, Matheus de Souza Gomes, Laurence Rodrigues do Amaral, Paulo Guilherme de Oliveira Salles

**Affiliations:** 1 Instituto Mário Penna Belo Horizonte MG Brazil Instituto Mário Penna, Belo Horizonte, MG, Brazil.; 2 Universidade Federal de Uberlândia Laboratório de Bioinformática e Análises Moleculares Patos de Minas MG Brazil Laboratório de Bioinformática e Análises Moleculares, Universidade Federal de Uberlândia, Patos de Minas, MG, Brazil.

**Keywords:** Gastric neoplasms, Cluster analysis, Genetic heterogeneity, Genes, p53, Immunohistochemistry, Adenocarcinoma, Biomarkers

## Abstract

**Objective:**

Gastric adenocarcinoma remains an aggressive disease with a poor prognosis, as evidenced by a 5-year survival rate of approximately 31%. The histological classifications already proposed do not accurately reflect the high biological heterogeneity of this neoplasm, particularly in diverse populations, and new classification systems using genetic markers have recently been proposed. Following these newly proposed models, we aimed to assess the cluster distribution in a Brazilian cohort. Furthermore, we evaluated whether the inclusion of other clinical and histological parameters could enhance the predictive value.

**Methods:**

We used a previously described four-immunohistochemistry/EBER-ISH marker to classify a cohort of 30 Brazilian patients with gastric adenocarcinoma into five different clusters and compared the distribution with other genetically diverse populations. Furthermore, we used artificial intelligence methods to evaluate whether other clinical and pathological parameters could improve the results of the methodology.

**Results:**

Disclosing the genetic variability between populations, we observed a more balanced distribution of the aberrant/normal p53 ratio (0.6) between patients negative for the other markers tested, unlike previous studies with Asian and North American populations. In addition, decision tree analysis reinforced the efficiency of these new classifications, as the stratification accuracy was not altered with or without additional data.

**Conclusion:**

Our study underscores the importance of local research in characterizing diverse populations and highlights the complementary role of molecular biomarkers in personalized medicine for gastric adenocarcinoma, enhancing diagnostic accuracy and potentially improving survival rates.

## In Brief

Queiroz et al. showed that the application of cluster methodology for classifying gastric cancer is suitable and efficient within a Brazilian cohort, which is known for its population heterogeneity. The study highlighted the potential utilization of this method within public health services due to its low-cost, presenting a viable means to improve the diagnosis and prognosis of gastric cancer.

## Highlights

◼ Our Brazilian cohort with gastric cancer has a distinct distribution between mutated and normal p53.
◼ New genetic marker-based classifications improve gastric cancer diagnosis accuracy.
◼ Machine learning integration enhances predictive value in gastric cancer diagnosis.
◼ Molecular biomarkers complement clinical decisions, advancing personalized medicine.

## Graphical abstract

**Figure:**
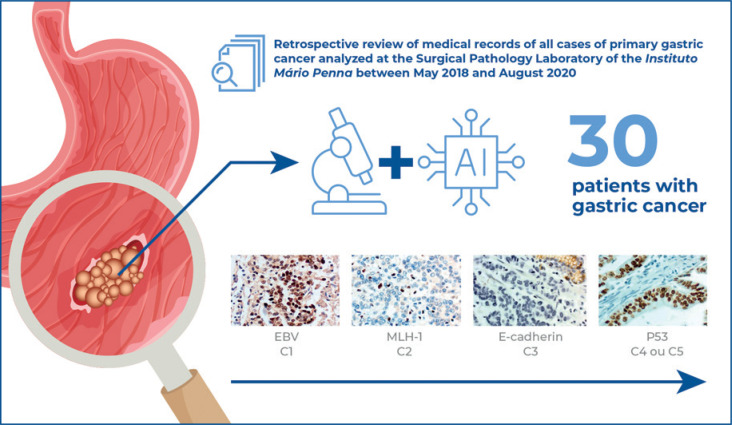

## INTRODUCTION

Gastric cancer (GC) is the fifth most common type of malignancy, with more than 1 million new cases per year worldwide.^(1–3)^ Despite improvements in diagnostic and therapeutic approaches, the prognosis of patients with GC remains unclear. It is the third deadliest cancer, with 783,000 deaths annually and a 5-year survival rate of only 31%.^(4)^ Historically, GC classification has relied on microscopic features associated with specific marker expression. Over the years, a better understanding of the genetic and molecular aspects of GC has resulted in new subtype stratification systems, demanding more efficient classification tools with clinical applicability, such as prognostic correlations and targeted therapies.

Laurén's classification, one of the first and most widely used GC classification systems, divides gastric adenocarcinomas into intestinal, diffuse, or mixed subtypes.^(5)^ However, this classification does not entirely consider the heterogeneous nature of diseases. Consequently, it has a poor association with tumor response to treatment and prognosis, failing to identify patients who can benefit from new therapies. The World Health Organization (WHO) classification^(6)^ is more complex. Relying on more precise histological patterns, it considers all rare GC subtypes that were not previously included in Laurén's classification. Nevertheless, it lacks clinical applicability because distinct histological subgroups are generally not implicated in different outcomes.^(7)^

To better understand the molecular and genetic aspects of GC, The Cancer Genome Atlas (TCGA) and the Asian Cancer Research Group (ACRG) used next-generation sequencing data to identify dysregulated pathways and candidate gene mutations.^(8,9)^ These mutations have emerged as possible molecular biomarkers and may contribute to drug development for specific subsets of GC. Some of the identified molecular markers include ErbB2 (Her-2), CDH1, and mismatch repair (MMR) genes. Although they better reflect tumor heterogeneity and correlate subgroups with targeted treatments and prognoses, these new approaches lack clinical applicability, mainly because of the use of sophisticated and expensive technologies, which limit reproducibility in the clinical setting.

To overcome technical difficulties in the clinical care of patients with GC and associate molecular profiles with treatment and prognosis, more straightforward techniques, such as immunohistochemistry (IHC), remain the gold standard and cost-effective alternative. For instance, patients with microsatellite instability and Epstein-Barr virus (EBV) infection are known to express PD-L1,^(10,11)^ which makes them potentially eligible for chemotherapy using immune checkpoint inhibitors, such as nivolumab^(12,13)^ and pembrolizumab.^(14)^ Other important biomarkers recently highlighted in GC include fibroblast growth factor receptor-2 (FGFR2) and Claudin 18.2 (CLDN18.2). Mutations in FGFR2 are present in approximately 4% of cases and are associated with a worse prognosis in GC.^(15,16)^ CLDN18.2 is exclusively expressed in differentiated epithelial cells of the gastric mucosa in primary GC.^(17)^ A recent study showed that therapy using the anti-CLDN18.2 chimeric monoclonal antibody, zolbetuximab, in combination with first-line chemotherapy provides significant survival benefits for patients with advanced GC.^(18)^

As an alternative/complement for Laurén's and WHO classification, and considering the tumor molecular aspects, Setia et al. proposed a method to segregate patients with GC into five clusters utilizing IHC.^(19)^ Cluster 1 (C1) is specifically for patients positive for EBV, with other markers not interfering in this classification. Patients negative for EBV and with a loss of MLH1 expression are classified into cluster 2 (C2), independent of E-cadherin (ECAD) and p53 expression. Both clusters have a better prognosis, and C1 patients usually benefit from immunotherapy.^(20)^ In the case of the absence of EBV and normal MLH1 expression, patients with aberrant ECAD expression (mutated or absent) belong to cluster 3 (C3), which has an unfavorable prognosis. p53 is considered for classification only if the previous parameters are normal. In this case, aberrant p53 expression determines cluster 4 (C4), while its normal expression defines cluster 5 (C5).

Considering the genetic heterogeneity across populations,^(20,21)^ particularly the heterogeneity within the Brazilian population for previously described cancer markers,^(22)^ it is pertinent to evaluate the performance of Setia et al. proposed classification in a Brazilian cohort.^(19)^

## OBJECTIVE

To assess the cluster distribution in a cohort of 30 Brazilian patients in comparison with other genetically diverse populations, and to evaluate whether the inclusion of other clinical and histological parameters yielded a better predictive value.

## METHODS

### Case selection and pathological diagnosis

We identified, selected, and evaluated 30 surgical cases of primary GC, representing approximately 20% of the annual number of cases during the evaluation period. Clinical and pathological data (age, sex, tumor histology and topography, invasion level, lymph node invasion, and pTNM stage) were obtained from medical records and used for patient classification, together with Laurén's criteria.

After approval by the Institutional Ethics Committee of Associação *Mário Penna* (CAAE: 39672920.2.0000.5121; #4.465.746), a retrospective chart review was conducted on all primary GC cases analyzed at the *Instituto Mário Penna* Surgical Pathology Lab between May 2018 and August 2020.

### Immunohistochemistry

All specimens were contained in paraffin-embedded blocks, which were sectioned into 3-4*μ*m slices for standard immunohistochemical staining of neoplastic cells. Mouse monoclonal antibodies directed against p53 (Leica, DO-7, ready-to-use), MLH1 (Leica-Biocore, E305, 1:50), and ECAD (Leica, 36B5, ready-to-use) were used. For EBV, EBER-ISH (Leica, BOND EBER Probe, ready-to-use) was used, and each reaction included negative and positive controls. The data obtained were used to identify and stratify patients into one of the five clusters proposed by Setia et al.^(19)^

### Statistical analysis

Data analysis was performed using GraphPad Prism^®^ 8.0 statistical software (GraphPad Software, Inc., San Diego, USA), using the χ^2^ test on a contingency table to evaluate the association between patient features and cluster classification. Differences were considered statistically significant at p<0.05.

### Development and training of gastric cancer classifier algorithms

Decision trees were built using WEKA software (Waikato Environment for Knowledge Analysis, version 3.6.11, University of Waikato, New Zealand) to classify patients with GC into one of five clusters based on clinicopathological features and IHC data. Leave-one-out cross-validation (LOOCV) was applied to estimate classification accuracy and test the generalizability of the model.

## RESULTS

A total of 30 patients were included in this study. The median diagnosis age was 61.5 years, and almost two-thirds of the patients were male (63.3%). Laurén's intestinal-type tumors were the most frequent (36.7%). Considering only the patients with available information (23/30), most cases were positive for lymphovascular (77.3%), perineural (78.3%), or lymph node invasion (63.6%). Five patients (21.7%) tested negative for all three features. Invasion of the subserosal layer was diagnosed in 73.9% of the cases. The clinical and pathological features of the patients are summarized in table 1. No statistically significant association was observed between clinicopathological features and cluster distribution.

**Table 1:** Clinicopathological features of patients with gastric carcinoma

Features		C1 (EBV)	C2 (MLH1)	C3 (ECAD)	C4 (Aberrant p53)	C5 (Normal p53)	n (%)	p value
n (%)		1 (3.3)	5 (16.7)	6 (20.0)	7 (23.3)	11 (36.7)	30 (100.0)	0.462
Median age		77.0	73.0	59.0	59.0	61.0	61.5
Sex								
	Female	0 (0.0)	4 (80.0)	2 (33.3)	1 (14.3)	4 (36.4)	11 (36.7)	0.187
	Male	1 (100.0)	1 (20.0)	4 (66.7)	6 (85.7)	7 (63.6)	19 (63.3)	
Tumor topography								
	Antrum-pylorus	1 (100.0)	1 (20.0)	0 (0.0)	1 (14.3)	3 (27.3)	7 (23.3)	0.769
	Body-fundus	0 (0.0)	2 (40.0)	2 (40.0)	3 (42.9)	7 (63.6)	14 (46.7)
	Cardia	0 (0.0)	0 (00.0)	0 (00.0)	1 (14.3)	1 (9.1)	2 (6.7)
	N/A	0 (0.0)	2 (40.0)	3 (60.0)	2 (28.6)	0 (0.0)	7 (23.3)
Laurén's type								
	Diffuse	0 (0.0)	1 (20.0)	5 (83.3)	1 (14.3)	2 (18.2)	9 (30.0)	0.105
	Intestinal-type	0 (0.0)	1 (20.0)	0 (0.0)	4 (57.1)	6 (54.5)	11 (36.7)	
	Mixed	1 (100.0)	3 (60.0)	1 (16.7)	2 (28.6)	3 (27.3)	10 (33.3)	
Lymphovascular invasion								
	Absent	0 (0.0)	1 (20.0)	0 (0.0)	0 (0.0)	3 (27.3)	5 (16.7)	0.632
	Present	1 (100.0)	2 (40.0)	3 (60.0)	4 (57.1)	7 (63.6)	17 (56.7)
	N/A	0 (0.0)	2 (40.0)	2 (40.0)	3 (42.9)	1 (9.1)	8 (26.7)	
Perineural invasion								
	Absent	0 (0.0)	1 (20.0)	1 (16.7)	0 (0.0)	3 (27.3)	5 (16.7)	0.583
	Present	1 (100.0)	2 (40.0)	3 (50.0)	5 (71.4)	7 (63.6)	18 (60.0)	
	N/A	0 (0.0)	2 (40.0)	2 (33.3)	2 (28.6)	1 (9.1)	7 (23.3)	
Lymph node invasion								
	Absent	0 (0.0)	1 (20.0)	1 (16.7)	2 (28.6)	4 (36.4)	8 (26.7)	0.848
	Present	1 (100.0)	2 (40.0)	2 (33.3)	3 (42.9)	6 (54.5)	14 (46.7)	
	N/A	0 (0.0)	2 (40.0)	3 (50.0)	2 (28.6)	1 (9.1)	8 (26.7)	
pT stage (invasion level)								
	T1	0 (0.0)	0 (0.0)	0 (0.0)	0 (0.0)	2 (18.2)	2 (6.7)	0.580
	T2	0 (0.0)	0 (0.0)	1 (16.7)	0 (0.0)	1 (9.1)	2 (6.7)	
	T3	1 (100.0)	3 (60.0)	2 (33.3)	5 (71.4)	7 (63.6)	18 (60.0)	
	T4	0 (0.0)	0 (0.0)	1 (16.7)	0 (0.0)	0 (0.0)	1 (3.3)	
	N/A	0 (0.0)	2 (40.0)	2 (33.3)	2 (28.6)	1 (9.1)	7 (23.3)	
pN stage								
	N0	0 (0.0)	0 (0.0)	1 (16.7)	2 (28.6)	4 (36.4)	7 (23.3)	0.217
	N1	0 (0.0)	0 (0.0)	0 (0.0)	0 (0.0)	3 (27.3)	3 (10.0)	
	N2	1 (100.0)	0 (0.0)	1 (16.7)	1 (14.3)	1 (9.1)	4 (13.3)	
	N3	0 (0.0)	3 (60.0)	1 (16.7)	2 (28.6)	2 (18.2)	8 (26.7)	
	NX	0 (0.0)	0 (0.0)	1 (16.7)	0 (0.0)	0 (0.0)	1 (3.3)	
	N/A	0 (0.0)	2 (40.0)	2 (33.3)	2 (28.6)	1 (9.1)	7 (23.3)	
pM stage								
	M0	1 (100.0)	3 (60.0)	3 (50.0)	5 (71.4)	10 (90.9)	22 (73.3)	0.290
	M1	0 (0.0)	0 (0.0)	1 (16.7)	0 (0.0)	0 (0.0)	1 (3.3)	
	N/A	0 (0.0)	2 (40.0)	2 (33.3)	2 (28.6)	1 (9.1)	7 (23.3)	

N/A: not available.

**Figure 1:**
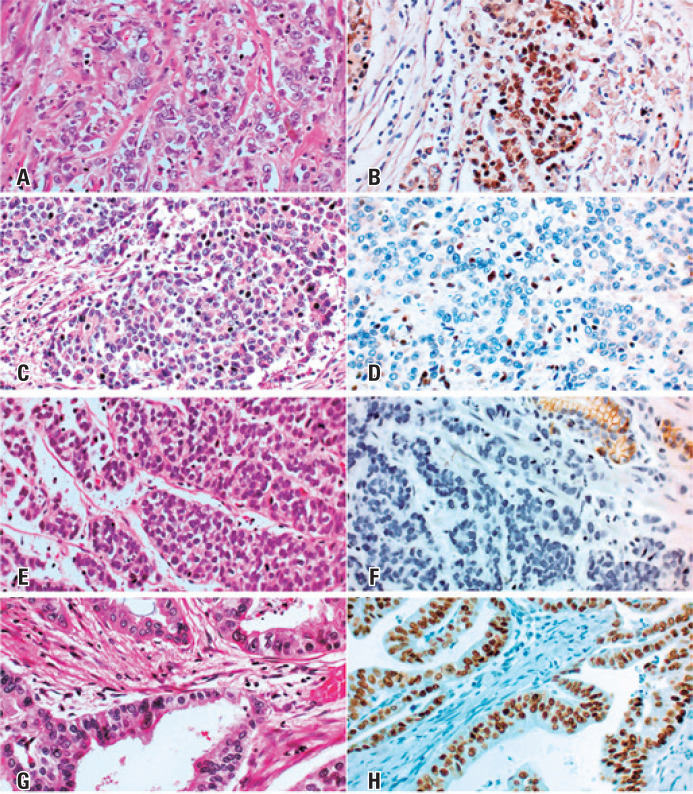
Histological results for gastric cancer classification. Left column (A, C, E, G): hematoxylin-eosin, 400×. Right column: immunohistochemistry (B: EBV (EBER-IHS); D: MLH1; F: ECAD; H: p53), 400×

Immunohistochemistry analyses of tumor samples for the biomarkers EBV, MLH1, p53, and ECAD were used to stratify the patients into the five clusters proposed by Setia et al.^(19)^ (Figure 1). More than one-third of the patients (36.7%) were classified as C5, characterized by p53 normal expression and the absence of the other tested markers. C2, C3, and C4 were roughly equally distributed, corresponding to 16.7%, 20.0%, and 23.3% of the patients, respectively (Table 2). Only one patient was positive for EBV and was classified as C1.

**Table 2:** Gastric cancer studies using cluster parameters in different populations

Population	C1 (EBV) %	C2 (MLH1) %	C3 (ECAD) %	C4 (Aberrant p53) %	C5 (Normal p53) %	n	Reference
North American	5	16	21	51	7	146	Setia et al., 2016^(19)^
Asian	7	7	15	49	22	349	Ahn et al., 2017^(20)^
Brazilian	10	18	7	35	30	287	Ramos et al., 2021^(25)^
Current study	3	17	20	23	37	30	

**Figure 2:**
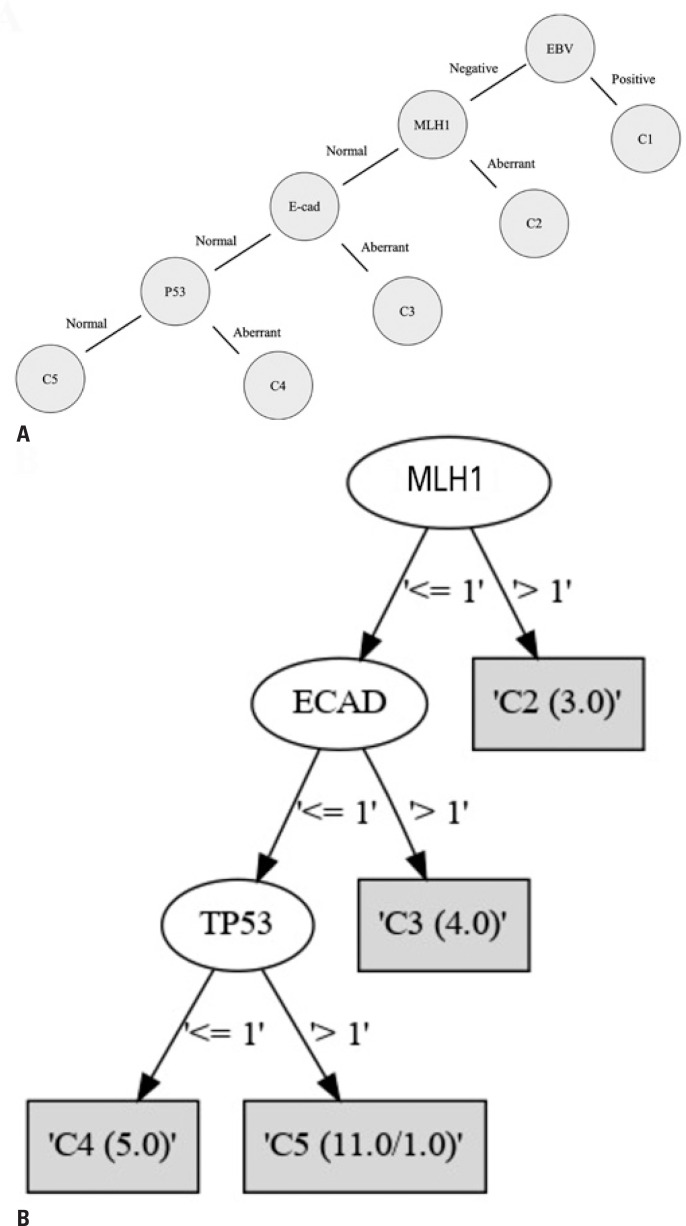
Gastric cancer classification parameters. (A) Cluster classification according to the expression of EBV, MLH1, ECAD, and p53. Setia et al. showed that patients with positive EBV expression, independent of other evaluated parameters, are classified as C1. Patients with a loss of MLH1 expression and who are negative for EBV, independent of the other parameters, are classified as C2. Patients with a loss of ECAD expression, who maintain normal MLH1 expression and are negative for EBV, are classified as C3. Patients expressing mutated p53, with the absence of EBV and normal expression of ECAD and MLH1, are classified as C4. Finally, patients that are negative for EBV with normal expression of MLH1, ECAD, and p53 wild type are classified as C5; (B) The classification algorithm confirmed the efficiency of cluster classification for separating gastric cancer groups without additional clinical parameters

Decision tree analysis was used to evaluate whether the inclusion of other clinical and histological parameters yielded a better predictive value. Patients with two or more missing data points were excluded, and the remaining 23 patients were considered. The clusters suggested by Setia et al.^(19)^ were tested using an algorithm with the additional data described in table 1. Stratification accuracy was not altered with or without additional data, highlighting the efficient classification proposed by Setia et al.^(19)^ The algorithm correctly classified 95.7% (22/23) of the samples tested in both the training and LOOCV sets (Figure 2). The use of other clinical and pathological parameters as possible classifiers did not yield improved results (Table 3).

**Table 3:** Accuracy of the classifiers using different features

Classifier	Features	Full training %	LOOCV %
Cluster	Clinicopathological + IHQ markers	95.65 (22/23)	95.65 (22/23)
Cluster	Clinicopathological	60.87 (14/23)	17.39 (4/23)
Invasion level	Clinicopathological + IHQ markers	86.96 (22/23)	65.22 (15/23)
Laurén	Clinicopathological	73.91 (17/23)	43.48 (10/23)
Laurén	Clinicopathological + IHQ markers	78.26 (18/23)	34.78 (8/23)

IHQ markers: Immunohistochemistry markers (EBER-ISH, MLH1, ECAD, and p53); LOOCV: Leave-one-out cross-validation.

## DISCUSSION

Gastric cancer is one of the most aggressive cancers, with one of the lowest overall survival rates worldwide.^(3)^ Despite the numerous novel chemotherapy regimens developed thus far, patient sensitivity to treatment varies, and some still fail to obtain satisfactory results. Thus, the accuracy of patient histological classification is crucial for estimating the prognosis and therapeutic strategies for GC and, consequently, improving the survival rate. Traditional classification methods, such as Laurén's and WHO classification, consider only histological patterns to subdivide patients into groups and do not fully represent the differences in treatment responses and prognosis. To overcome this limitation, Setia et al.^(19)^ proposed a straightforward method using cheaper and more broadly available technology to classify GC subtypes based on similar patterns found in TCGA and ACRG studies.

The effect of populational genetic diversity on the worldwide use of biomarkers is well known.^(23,24)^ In this study, we described the distribution of a cohort of 30 Brazilian patients into the five clusters proposed by Setia et al.^(19)^ The results showed that C1 was the least represented, with only one patient (3%) positive for EBV. A similar underrepresentation was observed in a North American population (5%).^(19)^ On the other hand, a higher C1 frequency has been described in another Brazilian cohort (10%).^(25)^ In an Asian population study, C1 and C2 were equally underrepresented compared to the other clusters (7% each).^(20)^ The higher incidence of microsatellite instability GCs (C2) in Western populations than in Asian populations has been described previously^(26–28)^ and corroborates our results, with 17% of patients classified as C2. Similar to an Asian study, we observed an increased median age in this group (73.0 years). However, our data differed in the prevalence of Laurén's intestinal-type subtype, with all three subtypes equally represented. Our data also corroborated the characteristics described in the Asian population for the C3 group, such as the prevalence of the Laurén's diffuse subtype (83.3%) and higher aggressiveness.^(20)^ In our cohort, this group harbored only one patient with distant metastasis.

The p53 aberrant expression rate in the absence of other IHC markers was the most discordant feature among the studies cited here, resulting in the discrepancies observed in the C4 and C5 distribution (Table 2). In the Asian population, C4 is twice as frequent as C5,^(20)^ and in the North American population, the frequency of C4 is more than seven times higher than that of C5.^(19)^ The Brazilian cohort described here, in turn, corroborates a more balanced distribution between the C4 and C5 groups, as observed previously,^(25)^ with Laurén's intestinal-type subtype present in more than half of these patients. Considering that the C5 group consisted of individuals who were negative for all other IHC markers analyzed, we could infer that nearly one-third of the patients were not stratified. Larger cohort studies may help to better characterize this group.

Subsequently, to select attributes that could permit better predictive value, we applied machine learning algorithm analysis. During the full training, the test included both clinical and pathological data. Algorithmic analysis confirmed that the accuracy obtained using the clusters proposed by Setia et al.^(19)^ was in accordance with our GC classification. The use of other clinical and pathological parameters as possible classifiers yielded no results, and the clusters were more suitable for GC classification.

Our study has some limitations, such as its small sample size, which may have influenced the differences observed between our cohort's cluster distribution and that of other Brazilian studies. In addition, almost one-fourth of the patients were not considered for the decision tree analysis because of missing clinicopathological data. In addition, other important markers, such as PD-L1, FGFR2, and CLDN18.2, were not evaluated. Finally, the short follow-up period prevented the evaluation of cluster categories associated with overall survival. Nonetheless, our data confirm the heterogeneity among the Brazilian population and reinforce that auxiliary data complementary to clinical information are necessary for accurate prognosis evaluation and clinical outcome prediction.

## CONCLUSION

In conclusion, our data corroborate the distinct pattern of aberrant p53 expression in Brazilian patients with gastric adenocarcinoma compared to other populations. Furthermore, this study highlights the importance of local research in characterizing specific groups of patients for personalized medicine and improving gastric adenocarcinoma survival rates. More studies with larger cohorts and long-term follow-up are essential to fully assess the utility of molecular analysis in gastric adenocarcinoma diagnosis and prognosis evaluation.

## References

[1] National Cancer Institute (NCI) (2021). TCGA. The Cancer Genome Atlas Program 2021 [updated 31/03/2021.

[2] Bray F, Ferlay J, Soerjomataram I, Siegel RL, Torre LA, Jemal A (2018). Global cancer statistics 2018: GLOBOCAN estimates of incidence and mortality worldwide for 36 cancers in 185 countries. CA Cancer J Clin.

[3] Ferlay J, Ervik M, Lam F, Colombet M, Mery L, Piñeros M (2020). Global Cancer Observatory: Cancer Today.

[4] Rawla P, Barsouk A (2019). Epidemiology of gastric cancer: global trends, risk factors and prevention. Prz Gastroenterol.

[5] Laurén P (1965). The Two Histological Main Types of Gastric Carcinoma: Diffuse and So-Called Intestinal-Type Carcinoma. An Attempt at a Histo-Clinical Classification. Acta Pathol Microbiol Scand.

[6] Nagtegaal ID, Odze RD, Klimstra D, Paradis V, Rugge M, Schirmacher P, Washington KM, Carneiro F, Cree IA, WHO Classification of Tumours Editorial Board (2020). The 2019 WHO classification of tumours of the digestive system. Histopathology.

[7] Sanjeevaiah A, Cheedella N, Hester C, Porembka MR (2018). Gastric Cancer: Recent Molecular Classification Advances, Racial Disparity, and Management Implications. J Oncol Pract.

[8] Cristescu R, Lee J, Nebozhyn M, Kim KM, Ting JC, Wong SS (2015). Molecular analysis of gastric cancer identifies subtypes associated with distinct clinical outcomes. Nat Med.

[9] Cancer Genome Atlas Research Network (2014). Comprehensive molecular characterization of gastric adenocarcinoma. Nature.

[10] Böger C, Behrens HM, Mathiak M, Krüger S, Kalthoff H, Röcken C (2016). PD-L1 is an independent prognostic predictor in gastric cancer of Western patients. Oncotarget.

[11] Cho YA, Lee H, Kim DG, Kim H, Ha SY, Choi YL (2021). PD-L1 Expression Is Significantly Associated with Tumor Mutation Burden and Microsatellite Instability Score. Cancers (Basel).

[12] Janjigian YY, Shitara K, Moehler M, Garrido M, Salman P, Shen L (2021). First-line nivolumab plus chemotherapy versus chemotherapy alone for advanced gastric, gastro-oesophageal junction, and oesophageal adenocarcinoma (CheckMate 649): a randomised, open-label, phase 3 trial. Lancet.

[13] Kelly RJ, Ajani JA, Kuzdzal J, Zander T, Van Cutsem E, Piessen G, Mendez G, Feliciano J, Motoyama S, Lièvre A, Uronis H, Elimova E, Grootscholten C, Geboes K, Zafar S, Snow S, Ko AH, Feeney K, Schenker M, Kocon P, Zhang J, Zhu L, Lei M, Singh P, Kondo K, Cleary JM, Moehler M, CheckMate 577 Investigators (2021). Adjuvant Nivolumab in Resected Esophageal or Gastroesophageal Junction Cancer. N Engl J Med.

[14] Sun JM, Shen L, Shah MA, Enzinger P, Adenis A, Doi T, Kojima T, Metges JP, Li Z, Kim SB, Cho BC, Mansoor W, Li SH, Sunpaweravong P, Maqueda MA, Goekkurt E, Hara H, Antunes L, Fountzilas C, Tsuji A, Oliden VC, Liu Q, Shah S, Bhagia P, Kato K, KEYNOTE-590 Investigators (2021). Pembrolizumab plus chemotherapy versus chemotherapy alone for first-line treatment of advanced oesophageal cancer (KEYNOTE-590): a randomised, placebo-controlled, phase 3 study. Lancet.

[15] Ooki A, Yamaguchi K (2021). The beginning of the era of precision medicine for gastric cancer with fibroblast growth factor receptor 2 aberration. Gastric Cancer.

[16] Schrumpf T, Behrens HM, Haag J, Krüger S, Röcken C (2022). FGFR2 overexpression and compromised survival in diffuse-type gastric cancer in a large central European cohort. PLoS One.

[17] Rohde C, Yamaguchi R, Mukhina S, Sahin U, Itoh K, Türeci Ö (2019). Comparison of Claudin 18.2 expression in primary tumors and lymph node metastases in Japanese patients with gastric adenocarcinoma. Jpn J Clin Oncol.

[18] Sahin U, Türeci Ö, Manikhas G, Lordick F, Rusyn A, Vynnychenko I (2021). FAST: a randomised phase II study of zolbetuximab (IMAB362) plus EOX versus EOX alone for first-line treatment of advanced CLDN18.2-positive gastric and gastro-oesophageal adenocarcinoma. Ann Oncol.

[19] Setia N, Agoston AT, Han HS, Mullen JT, Duda DG, Clark JW (2016). A protein and mRNA expression-based classification of gastric cancer. Mod Pathol.

[20] Ahn S, Lee SJ, Kim Y, Kim A, Shin N, Choi KU (2017). High-throughput Protein and mRNA Expression-based Classification of Gastric Cancers Can Identify Clinically Distinct Subtypes, Concordant With Recent Molecular Classifications. Am J Surg Pathol.

[21] Lei Z, Tan IB, Das K, Deng N, Zouridis H, Pattison S (2013). Identification of molecular subtypes of gastric cancer with different responses to PI3-kinase inhibitors and 5-fluorouracil. Gastroenterology.

[22] Ferlay J, Colombet M, Soerjomataram I, Mathers C, Parkin DM, Piñeros M (2019). Estimating the global cancer incidence and mortality in 2018: GLOBOCAN sources and methods. Int J Cancer.

[23] Wojcik GL, Graff M, Nishimura KK, Tao R, Haessler J, Gignoux CR (2019). Genetic analyses of diverse populations improves discovery for complex traits. Nature.

[24] Sirugo G, Williams SM, Tishkoff SA (2019). The Missing Diversity in Human Genetic Studies. Cell.

[25] Ramos MF, Pereira MA, de Mello ES, Cirqueira CD, Zilberstein B, Alves VA (2021). Gastric cancer molecular classification based on immunohistochemistry and in situ hybridization: analysis in western patients after curative-intent surgery. World J Clin Oncol.

[26] Noguchi Y, Yoshikawa T, Tsuburaya A, Motohashi H, Karpeh MS, Brennan MF (2000). Is gastric carcinoma different between Japan and the United States?. Cancer.

[27] Ohtsu A, Yoshida S, Saijo N (2006). Disparities in gastric cancer chemotherapy between the East and West. J Clin Oncol.

[28] Davis PA, Sano T (2001). The difference in gastric cancer between Japan, USA and Europe: what are the facts? what are the suggestions?. Crit Rev Oncol Hematol.

